# Healthcare professionals’ knowledge and practices in managing ototoxicity in children with cancer

**DOI:** 10.4102/sajcd.v71i1.1064

**Published:** 2024-11-25

**Authors:** Kajal Ramnarian, Jessica Paken

**Affiliations:** 1 Department of Audiology, Faculty of Health Sciences, University of KwaZulu-Natal, Durban, South Africa

**Keywords:** cancer, hearing loss, paediatrics, ototoxicity, knowledge, practices, platinum-based chemotherapy

## Abstract

**Background:**

Platinum-based chemotherapy poses a risk of ototoxic hearing loss, the effects of which can be devastating in paediatrics with cancer. Childhood hearing loss significantly impacts speech and language acquisition, and educational, psychosocial and emotional development, consequently negatively impacting quality of life. Adequate knowledge and effective management by healthcare professionals in the team managing paediatrics with cancer are, therefore, pivotal to mitigating the severity and impact on quality of life.

**Objectives:**

To describe the knowledge and practices of healthcare professionals on the management of ototoxic hearing loss in children receiving platinum-based chemotherapy drugs.

**Method:**

Using a descriptive survey design, data were collected from self-administered questionnaires completed by 74 healthcare professionals from two hospitals in KwaZulu-Natal, South Africa.

**Results:**

While 45 participants (60.8%) identified ototoxicity as a side effect of platinum-based chemotherapeutic drugs, 43 (58.1%) identified dose, duration and mode of administration as risk factors, and 43 participants (72.9%) did not know the duration of an ototoxicity monitoring programme post-treatment. Fifty participants (68%) accurately identified most of their roles within the ototoxicity monitoring programme. Most participants (*n* = 73; 99%) did not fully adhere to Health Professions Council of South Africa (HPCSA) ototoxicity monitoring guidelines. However, a positive outcome was that 70 participants (94.6%) acknowledged the importance of the ototoxicity monitoring programme for children receiving platinum-based chemotherapy.

**Conclusion:**

The current study demonstrates a clear correlation between healthcare professionals’ practices and their level of knowledge. These findings underscore the importance of improving the knowledge base of healthcare professionals involved in ototoxicity monitoring programme to enhance their practices effectively.

**Contribution:**

This study identified areas requiring improvement in managing ototoxicity in this patient group, prompting the inclusion of ototoxicity training. This study supports audiologists in effectively implementing and overseeing ototoxicity monitoring programme.

## Introduction

The global prevalence of cancer cases is predicted to increase by 77% over the next two decades (World Health Organization [WHO], 2024); however, childhood cancer survival rates have improved to 80% in most developed countries (WHO, 2021) because of effective cancer treatments, such as radiotherapy and chemotherapy. In African communities, approximately 30% of children with cancer survive as compared to higher-income countries, mainly because of limited access to cancer screening, assessment and intervention services (Moeti, 2021, as cited in WHO, 2021). South Africa has tertiary hospitals with oncology units offering specialised oncology services; however, less than 50% of suspected cases are diagnosed, and those who are diagnosed are often diagnosed in advanced stages (Cancer Association of South Africa [CANSA], 2024), thus necessitating the use of potent chemotherapeutic drugs containing the chemical element platinum as part of their molecular structure.

While the platinum-based chemotherapeutic drugs, namely cisplatin, carboplatin and oxaliplatin, used individually or in conjunction with other therapies, are effective in treating many malignant tumours (Zhang et al., 2022), they have numerous toxic effects. Side effects of platinum-based chemotherapy drugs include nephrotoxicity, neurotoxicity, cardiotoxicity, haematological toxicities, hepatotoxicity, gastrointestinal toxicities and ototoxicity (Wheate et al., 2018). Ototoxicity is defined as the temporary or permanent impairment and tissue degeneration of the inner ear and neurons of the vestibulocochlear nerve caused by ototoxic drugs (Bisht & Bist, 2011). Permanent, bilateral high-frequency sensorineural hearing loss and tinnitus are common clinical features of platinum-based ototoxicity (Wheate et al., 2018). While the hearing loss is permanent, tinnitus may resolve after completing treatment (Paken et al., 2023). High-frequency (> 2 kHz) hearing loss causes difficulty in speech discrimination (Roland & Rutka, 2004). Other side effects include hyperacusis, aural fullness and vertigo and/or dizziness (Health Profession Council of South Africa [HPCSA], 2007).

Patients may consider hearing loss from ototoxicity to be a major barrier to their everyday activities and general quality of life even though the treatment of cancers is a major advantage (Theunissen et al., 2014). Furthermore, the acquisition of speech and language abilities by young children can be adversely affected by even mild hearing loss (Bess et al., 1998; Crandell & Smaldino, 2000; McFadden & Pittman, 2008). Severe or profound high-frequency hearing loss in children can significantly impact speech perception, speech production, speech intelligibility, language development and communication (Rajput et al., 2020). High-frequency speech phonemes and fricative phonemes help with phonological and morphological development (Spratford et al., 2017). The consequences of hearing loss in children are detrimental because language development usually occurs at a fast rate during childhood, and hearing loss during childhood can result in slow language acquisition. The impact of delayed speech and language development may be difficult to overcome even with amplification or speech therapy (Tomblin et al., 2015). Therefore, it is crucial to prioritise early detection and appropriate management of ototoxicity in children, necessitating the inclusion of an audiologist within the healthcare team.

Children require different management than adults because they face a higher risk when exposed to similar treatments (Al-Malky et al., 2015; Knight et al., 2005). Additionally, it is essential to counsel parents or caregivers about potential hearing and balance issues and gather information about the child’s speech, language and motor development during initial testing. Effectively managing platinum-based ototoxicity necessitates a collaborative approach involving multiple disciplines, including audiology, oncology, physiotherapy, pharmacy, otolaryngology, nursing, occupational therapy, paediatricians and psychology. The management of ototoxicity in children undergoing platinum-based chemotherapy may differ, which could result in an inadequate detection of ototoxicity and exacerbate the difficulties associated with controlling the consequences of hearing loss. A concerning number of children receiving cancer treatment may develop ototoxic hearing loss as a result of their platinum-based chemotherapy. Cisplatin-induced ototoxicity usually occurs in 60% of children (Nunez-Batalla et al., 2022). According to Spratford et al. (2017), the quality of life of children with ototoxic hearing loss was rated as poorer in areas of personal communication, self-independence and emotional well-being; yet the severity of this condition and its negative effects on a person’s quality of life may be reduced with appropriate management. Without adequate knowledge and training of healthcare professionals treating children on platinum-based chemotherapy drugs in identifying adverse drug reactions, the impact can be detrimental and irreversible.

While there are many national studies investigating ototoxicity, there are currently no studies on the knowledge and practices of healthcare providers on ototoxic hearing loss in children receiving platinum chemotherapeutic drugs in South Africa, thus providing the impetus for the current study. The current study focussed on describing the practices and knowledge of healthcare professionals in KwaZulu-Natal on ototoxic hearing loss in children receiving platinum chemotherapeutic drugs. This information may assist in identifying gaps in the management of ototoxicity in this patient population, which can be addressed through in-service training. It is further envisaged that the findings of this study may also help audiologists advocate for their role in the ototoxicity monitoring programme.

### Aim and objectives

The study aimed to describe healthcare professionals’ knowledge and practices on the management of ototoxic hearing loss in children receiving platinum-based chemotherapy drugs.

This study had the following objectives:

- To describe the healthcare professionals’ awareness of ototoxicity and the ototoxic effects of platinum-based chemotherapy drugs on the auditory system.
- To describe the healthcare professionals’ knowledge of their roles, the role of audiologists and other team members in an ototoxicity monitoring programme for children.
- To describe health care professionals’ practices in managing ototoxicity in children.
- To identify factors influencing the practice of health care professionals in managing ototoxicity in children.

## Research methods and design

### Study design

A descriptive survey design was employed in the current study.

### Setting

The study was conducted at two public hospitals offering oncology services in KwaZulu-Natal, South Africa. These two hospitals were selected because they are the only two hospitals in KwaZulu-Natal with designated oncology wards rendering all oncological services to children.

### Study population

The following study population was selected because these healthcare professionals form part of the interdisciplinary team within an ototoxic monitoring programme (HPCSA, 2018): (1) ears, nose and throat specialist (ENT), (2) paediatricians, (3) paediatric oncologists, (4) audiologists, (5) registered nurses, (6) pharmacists, (7) speech therapists, (8) physiotherapists and (9) clinical psychologists.

### Sampling technique

Maximum variation sampling was used because the researcher wanted to describe the knowledge and practices of the different healthcare professionals specialising in paediatric oncology. The various healthcare professionals in this study were explicitly selected using the following inclusion criteria: (1) participants must be registered with relevant health/medical councils and (2) participants must be working in the paediatric oncology ward.

### Sample size

A sample size of 90 participants was required to achieve a 95% confidence level with a 5% error margin and a 5% level of significance. In the current study, there was an 82% response rate, that is, 74 healthcare professionals participated in the study.

### Data collection instrument

Data were obtained by means of an anonymous and self-administered questionnaire, which consisted mainly of close-ended and limited open-ended questions (refer to Online Appendix 1). The questionnaire consists of five sections comprising 31 questions in total: Section 1 (biographical information), Section 2 (knowledge of ototoxicity and ototoxic effects of platinum-based chemotherapeutic drugs and Section 5 (factors influencing the practices of healthcare professionals) contain questions applicable to all healthcare professionals selected for the study. Section 3 (inter-professional roles within the ototoxicity monitoring programme) contains some common questions and questions specific to each of the different healthcare professionals. As each healthcare profession’s roles differ, this section contains questions specific to roles within the selected fraternities. Section 4 includes questions relating to the practices of each selected profession; therefore, it contains different questions. Questionnaires were in English only since English is the medium of instruction at universities and Technikons in South Africa (Ngidi & Mncwango, 2022). The questionnaire was adapted from existing literature, that is, Harrison (2018) and Al-Malky (2014), as well as guidelines from American Academy of Audiology (2009).

### Data collection procedure

Following receipt of ethical clearance from the Institution’s Ethics Committee and gatekeeper permission, the researcher communicated with management at the selected sites to arrange the delivery of the questionnaires. The Head of Department (HOD) was emailed every 2 weeks to remind the participants to complete the questionnaire. All healthcare staff in each department were provided with the information document, consent form and questionnaire by the HOD, and those wanting to participate signed the informed consent document, which was submitted together with the completed questionnaire in a closed collection box placed in the department. A pilot study was conducted with 10 participants prior to the main study. The pilot study consisted of at least one participant from each category. These participants were not included in the main study. The following adaptations were made following the pilot study; the suggestions for the questionnaire included tables that should be restricted to one page. This suggestion was accommodated where possible, and adding the option ‘sometimes’ to the yes/no options was included. Upon completion of data collection, all healthcare personnel received a pamphlet developed by the researcher on ototoxicity and the ototoxicity monitoring programme to reinforce their knowledge of the topic area and clear any misconceptions. The data collection process was conducted over 3 months.

### Data analysis

Data analysis was carried out with the assistance of a statistician. The quantitative data analysis was conducted in Statistical Package for the Social Sciences (SPSS) (v 29.0) (IBM Corporation, Armonk, New York, United States). The descriptive analysis was presented as percentages, counts, frequency tables, cross-tabulation tables and graphical displays. The relationship between knowledge and practices was established using multinomial logistic regression. Inferential tests used in this study were the chi-square goodness of fit test, the chi-square test of independence, Pearson’s correlation and the independent Kruskal-Wallis test with analysis of variance (ANOVA). A *p*-value of less than 0.05 was considered statistically significant. Open-ended questions were analysed thematically.

The knowledge scores were determined by stating the number of accurate answers by each participant as a percentage. Answers on practices were analysed with the use of contingency tables. The score brackets of not achieved (0% – 29%), elementary achievement (30% – 39%), moderate achievement (40% – 49%), adequate achievement (50% – 59%), substantial achievement (60% – 69%), meritorious achievement (70–79%) and outstanding achievement (80% – 100%) were used to grade the percentage scores of knowledge. The grading system was developed in correlation with South Africa’s National Protocol for Assessments, Grade R-12 examinations, chapter 5: recording and reporting of learner performance (Department of Basic Education, n.d).

### Ethical considerations

Ethical clearance was obtained from the University of KwaZulu-Natal Biomedical Ethics Committee (reference no. BREC/00002526/2021). Participants provided informed consent, and the study adhered to the principles of the Declaration of Helsinki. Non-maleficence was maintained as no participants were harmed in this study. The principles of beneficence were adhered to. Participants right to privacy was maintained as participation was voluntary and confidential. Data were obtained using an anonymous and self-administered questionnaire. Written consent from the participants was ascertained prior to conducting the study. In order to maintain anonymity, the completed questionnaires were placed in a closed collection box, numbers were assigned to completed questionnaires and participants were not required to reveal their identity. Participants were allowed to withdraw their consent from the study at any time and were informed of any changes made. Healthcare professionals who declined participation were not treated with prejudice. The current study upheld complete anonymity, as the researcher could not make participant-data connections. All participants were treated fairly as each participant received an information sheet, consent form and questionnaire, and HODs from each department received pamphlets to distribute to all the staff following the collection of the completed questionnaires.

## Results

### Participants demographics

As indicated in Table 1, most participants were nurses (*n* = 19; 25.7%), and only one (1.4%) was a paediatric oncologist. All participants offered services to children. Thirty-nine participants (54.9%) had more than 10 years of experience, and 71 (95.9%) participants studied in South Africa. With regard to studies or receiving training on the ear, hearing and ototoxicity, 26 participants (36.1%) indicated that they did not study or receive training. Furthermore, 23 (31.1%) participants indicated that the study on ear, hearing and ototoxicity was through undergraduate studies. Thirty-four (54.0%) participants indicated that their training was inadequate.

**TABLE 1:** Participant’s demographics.

Categories	*n*	%	*p*
**Profession**	-	-	< 0.001
Audiology	4	5.4	
Pharmacy	17	23.0	
Psychology	4	5.4	
Physiotherapy	12	16.2	
Ear, nose and throat specialist	14	18.9	
Nursing	19	25.7	
Paediatric Oncology	1	1.4	
Occupational Therapy	3	4.1	
Paediatrician	2	2.7	-
**To which group do you offer services?**	-	-	-
Children	74	100.0	-
**Number of years working as a professional**	-	-	< 0.001
0–1	7	9.9	
2–5	9	12.7	
6–10	16	22.5	
> 10	39	54.9	
**Number of years offering healthcare services to children**	-	-	0.982
0–1	16	24.6	
2–5	17	26.2	
6–10	15	23.1	
> 10	17	26.2	
**Study in South Africa**	-	-	< 0.001
No	2	2.7	
Yes	71	95.9	
**Study or receive training about the ear, hearing and ototoxicity**	-	-	0.018
No	26	36.1	
Yes	46	63.9	
**If Yes: (follow up to the above question-multiple responses)**	-	-	-
Undergraduate studies	23	31.1	-
Postgraduate studies	14	18.9	-
Orientation	2	2.7	-
In-service training	6	8.1	-
On-the-job training	13	17.6	-
Other	5	6.8	-
**Training or studies on the ear, hearing or ototoxicity were adequate**	-	-	0.529
No	34	54.0	-
Yes	29	46.0	-

A *p*-value of less than 0.05 was considered statistically significant. The profession, number of years working as a professional and place of study were statistically significant, indicating that these variables influence knowledge and practice.

#### Objective 1: To describe the healthcare professionals’ awareness of ototoxicity and the ototoxic effects of platinum-based chemotherapy drugs on the auditory system

**FIGURE 1:**
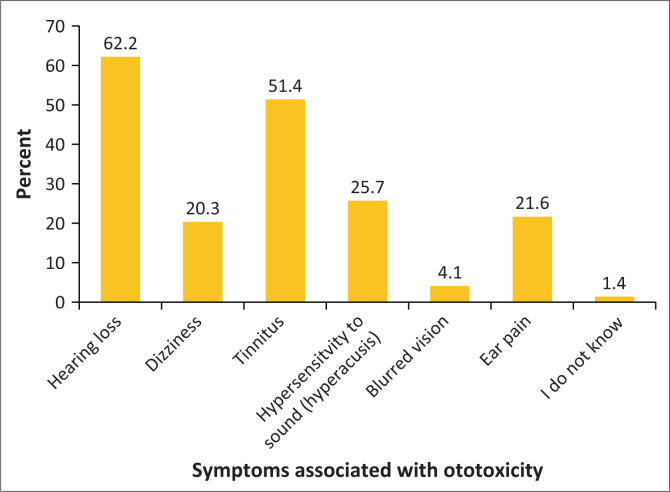
Participants’ knowledge of the symptoms associated with ototoxicity.

Forty-nine (66.2%) participants identified hearing loss, while 38 (51.4%) indicated tinnitus and 19 (25.7%) indicated hyperacusis as symptoms of ototoxicity, as indicated in Figure 1.

**FIGURE 2:**
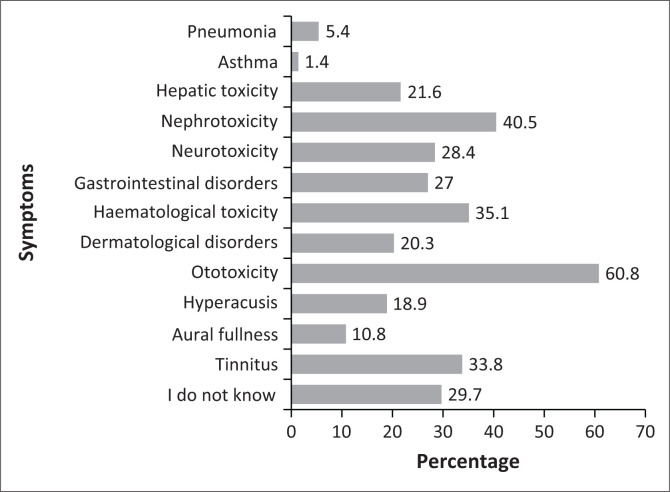
Participants’ knowledge of the side effects of platinum-based chemotherapeutic drugs.

As reflected in Figure 2, 45 participants (60.8%) recognised ototoxicity as a potential side effect of platinum-based chemotherapeutic drugs; however, this finding did not yield statistical significance (*p* = 0.063). Nephrotoxicity and haematological toxicity were correctly identified as associated side effects of platinum-based chemotherapy drugs by 30 (40.5%) and 26 (35.1%) of the participants, respectively. Eight (10.8%) and 25 (33.8%) participants indicated aural fullness and tinnitus, respectively, as side effects of platinum-based chemotherapy drugs.

**FIGURE 3:**
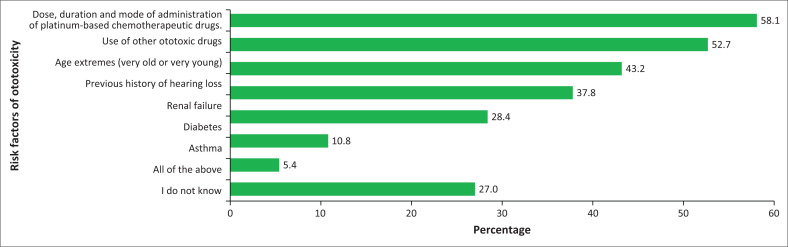
Participants’ understanding of risk factors for ototoxicity caused by platinum-based chemotherapeutic drugs.

Forty-three participants (58.1%) identified dose, duration and mode of administration as risk factors, while 39 participants (52.7%) identified the use of other ototoxic drugs and 32 (43.2%) identified age. However, it is worth noting that 20 participants (27.0%) indicated not knowing the risk factors of ototoxic hearing loss, as reflected in Figure 3.

#### Objective 2: To describe the healthcare professionals’ knowledge of their roles, the role of audiologists and other team members in an ototoxicity monitoring programme for children

Two-thirds of the respondents (67.7%) did not know if there was currently an ototoxicity monitoring programme at the institution where they were employed, while 13 participants (20.0%) indicated that there was one, 8 (12.3%) denied having one in place.

**FIGURE 4:**
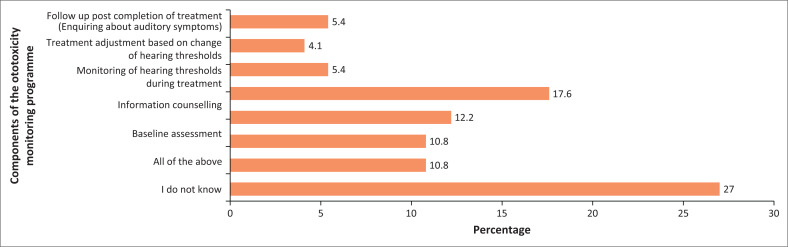
Components of the ototoxicity monitoring programme.

Twenty (27.0%) participants, as seen in Figure 4, did not accurately identify all the components of an ototoxicity monitoring programme. In comparison, only three participants (4.1%) knew that treatment adjustment based on changes in hearing was part of the ototoxicity monitoring programme.

Forty-three (72.9%) of the 59 participants who responded to the duration of the ototoxicity monitoring programme indicated not knowing the duration of an ototoxicity monitoring programme post-treatment (Table 2). In comparison, only 10 participants (16.9%) accurately indicated that the ototoxicity monitoring programme continues for up to 6 months following cessation of treatment.

**TABLE 2:** Duration of an ototoxicity monitoring programme post-treatment.

Duration	Frequency	%
Monitoring stops as soon as treatment is stopped or completed	2	3.4
Up to 6 months following cessation of treatment	10	16.9
Longer than 6 months post-treatment	4	6.8
I do not know	43	72.9
**Total**	**59**	**100.0**

Furthermore, 70 participants (94.6%) indicated that the ototoxicity monitoring programme is essential for children receiving platinum-based chemotherapy drugs, with 56 participants (77.8%) indicating that they do have a role within the programme, 12 (16.7%) uncertain and the remaining 4 (5.6%) responding negatively.

All audiologists (100.0%) accurately identified most of their roles within the ototoxicity monitoring programme; however, one (25.0%) participant did not identify raising awareness of ototoxic drugs as part of an audiologist’s responsibilities. One participant (25.0%) inaccurately indicated that adjusting the treatment regimen was part of the audiologist’s responsibilities. Eleven (64.7%) pharmacists accurately indicated that their role included raising awareness of the potential for ototoxicity of drugs prescribed; however, only seven (41.7%) included that suggesting otoprotective treatments were part of their scope of practice. All clinical psychologists (100.0%) correctly indicated that their role includes managing the patients’ psychological well-being when dealing with ototoxic impairment. In comparison, one psychologist (25.0%) did not indicate that their role also comprises offering counselling/support to the patient’s family members. Only 50.0% of physiotherapists indicated that their role within the ototoxicity monitoring programme includes providing inter-professional vestibular rehabilitation. The paediatricians accurately indicated their roles within the ototoxicity monitoring programme, which included assessing the patient for co-morbidities, initiating oncological treatment for patients, enquiring about auditory symptoms and requesting for audiological assessments in response to the auditory symptoms. Twelve (85.7%) ear, nose and throat (ENT) participants correctly indicated enquiring about auditory symptoms, and 10 (71.4%) correctly indicated requesting for audiological assessments in response to auditory symptoms. Twelve (63.2%) nurses accurately indicated that their role included monitoring ototoxic signs and symptoms and making appropriate referrals. In comparison, only six (31.6%) indicated providing informational counselling, and four (21.1%) inaccurately indicated vestibular rehabilitation as part of their responsibilities. The one paediatric oncologist participant accurately indicated her roles within an ototoxicity monitoring programme. These roles included assessing patients for co-morbidities and requesting for baseline assessments, initiating oncological treatment for patients, reviewing treatment of patients on platinum-based chemotherapeutic drugs in response to the identification of ototoxicity and enquiring about auditory symptoms.

Table 3 reflects the overall knowledge scores of ototoxicity ranging from ‘not achieved’ to ‘moderate’ according to the Department of Basic Education Assessment protocols.

**TABLE 3:** Overall knowledge scores of ototoxicity.

Knowledge of ototoxicity	Overall scores (%)	Achievement description
Risk factors of ototoxicity caused by platinum-based chemotherapeutic drugs	40.80	Moderate
Symptoms you think are associated with platinum-based ototoxicity	37.04	Elementary
Side effects of platinum-based chemotherapeutic drugs	31.24	Elementary
Components of the ototoxicity monitoring programme	9.25	Not achieved

Table 3 displayed a moderate level of knowledge on risk factors and an elementary level of knowledge on symptoms and side effects of ototoxicity among healthcare professionals.

#### Objective 3: To describe healthcare professionals’ practices in managing ototoxicity in children

Three audiologists (75.0%) reported informing the patient’s parents/caregiver about the ototoxic effects of platinum-based chemotherapeutic drugs (Table 4). All audiologists reported conducting otoscopy, tympanometry, acoustic reflex threshold testing and pure tone audiometry for baseline and follow-up assessments. However, only two (50.0%) reported conducting high-frequency audiometry, and one (25.0%) reported excluding distortion product otoacoustic emission test from the follow-up assessment. Three audiologists (75.0%) reported using an inter-professional approach. Two (50.0%) indicated inadequate training on ototoxicity and lack of resources as challenges within the ototoxicity monitoring programme. Three audiologists (4.1%) reported explaining to the family the ototoxic effects of platinum-based chemotherapeutic drugs. Only three (17.6%) audiologists indicated that an explanation of the ototoxic effects of chemotherapy drugs is provided before the start of treatment, and six (35.3%) reported sometimes explaining the effects before treatment commences.

**TABLE 4:** Audiologists’ management of children receiving platinum-based chemotherapy drugs.

Question	Options	Number	%
Do you inform the patient’s parents/caregiver of the ototoxic effects of platinum-based chemotherapeutic drugs?	Yes	3	75.0
	No	1	25.0
If yes, when do you provide informational counselling on the ototoxic effects of platinum-based chemotherapeutic drugs to patients/caregivers?	Before treatment	2	66.7
	Before treatment + After treatment has stopped/completed + While the patient is on treatment + When the patient starts experiencing symptoms	1	33.3
What tests are used to conduct hearing assessments on children?	Otoscopic examination	4	100.0
	Tympanometry	4	100.0
	Acoustic reflex threshold testing	4	100.0
	Distortion product otoacoustic emissions	4	100.0
	Transient evoked otoacoustic emission	0	0.0
	High-frequency Audiometry	2	50.0
	Auditory brainstem Response	3	75.0
	Auditory steady-state response	3	75.0
	Pure tone audiometry	4	100.0
What tests are used to conduct follow-up hearing assessments on children?	Otoscopic examination	4	100.0
	Tympanometry	4	100.0
	Acoustic reflex threshold testing	4	100.0
	Distortion product otoacoustic emissions	3	75.0
	Transient evoked otoacoustic emission	1	25.0
	High-frequency Audiometry	2	50.0
	Auditory brainstem Response	3	75.0
	Auditory steady-state response	3	75.0
	Pure tone audiometry	4	100.0
Do you use an inter-professional approach when treating patients on platinum-based chemotherapeutic drugs?	Yes	0	0.0
	No	3	75.0
What challenges do you face within the ototoxicity monitoring programme? You may select more than one option.	None	1	25.0
	Lack of resources	2	50.0
	Lack of time	1	25.0
	Lack of skills from healthcare professionals	0	0.0
	Inadequate training on ototoxicity	2	50.0
	Other (Participant’s comment: ‘Some children cannot leave the ward when on Rx as their immune system is too weak.’)	1	25.0

Twelve ENTs (86.0%) indicated referring children for audiological assessments, and 13 ENTs (92.9%) used the inter-professional approach when treating patients on platinum-based chemotherapeutic drugs. Thirteen nurses (68.4%) indicated referring patients for audiological assessment. Difficulty hearing was the most common indicator used to refer a patient (63.2%, *n* = 12), while only three (15.8%) used hypersensitivity to sound as a sign.

One (33.0%) occupational therapist referred patients for audiological assessments when needed, and one (33.0%) used the inter-professional approach. Four (40.0%) physiotherapists reported not conducting balance assessments on children receiving platinum-based chemotherapy drugs. Six physiotherapists (60.0%) indicated that a vestibular assessment/screening was conducted when the patient presented with signs of vestibular disorders. Two physiotherapists (20.0%) indicated that a vestibular assessment/screening was conducted after the first dose of platinum-based chemotherapeutic drugs. Two physiotherapists (20.0%) also indicated that they use an inter-professional approach when treating patients on platinum-based chemotherapeutic drugs, while six (60.0%) indicated that they sometimes use an inter-professional approach.

Two (50.0%) clinical psychologists (*n* = 2) indicated counselling patients/parents of patients on platinum-based chemotherapeutic drugs, with the majority (75.0%; *n* = 3) indicating the criteria used to determine when counselling should begin was when the patient starts to show signs of emotional disturbances. Fifty per cent of the psychologists use an inter-professional approach when treating patients on platinum-based chemotherapeutic drugs.

Only three pharmacists (18.2%) indicated that the ototoxic effects of platinum-based chemotherapy are explained to the patient’s family before the commencement of treatment, and six (35.3%) indicated that an inter-professional approach is utilised when treating patients on platinum-based chemotherapy.

The paediatric oncologist (*n* = 1;100.0%) indicated that all children on platinum-based chemotherapy are referred for audiological assessments. The paediatrician participants (*n* = 2; 100.0%) reported the criterion for referral to an ototoxicity monitoring programme to be ‘any child on platinum-based chemotherapy drugs’, and an inter-professional approach is utilised when treating patients on platinum-based chemotherapy.

#### Objective 4: To identify factors influencing the practice of healthcare professionals in managing ototoxicity in children

**FIGURE 5:**
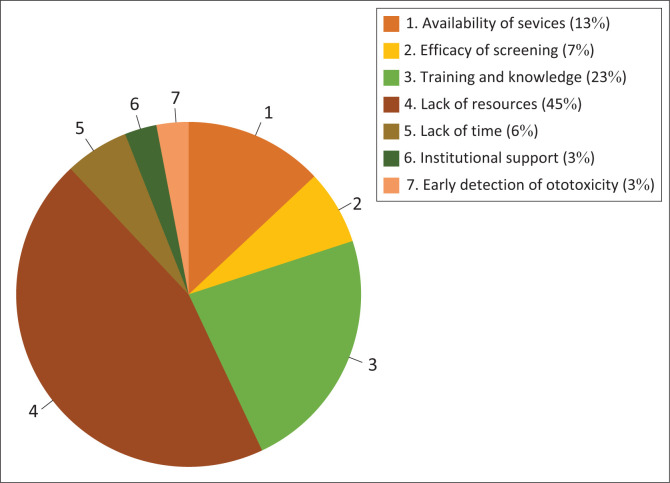
Factors influencing the practice of healthcare professionals in managing ototoxicity in children.

Fourteen (19.0%) healthcare professionals indicated that a lack of resources influences their practices in managing ototoxicity in children (Figure 5). Other factors included the availability of a multidisciplinary team, time constraints and a lack of knowledge of drugs and ototoxicity.

Audiologists, ENTs, physiotherapists and paediatrician identified shortage of staff as a challenge. Lack of commitment by staff was a challenge pointed out by the nurses, clinical psychologists and paediatric oncologists. Resource constraints were further identified as a problem reported by ENTs, audiologists, pharmacists, physiotherapists, clinical psychologists, paediatric oncologists and nurses (Table 5).

**TABLE 5:** Profession specific challenges.

Profession	Challenge
Ear, nose and throat specialist	Availability of audiological services, resource limitations, access to multiple disciplines and shortage of staff.
Audiologist	Staff shortage, lack of equipment and budget constraints.
Pharmacist	Time constraints, availability of resources, training and working in multidisciplinary team.
Physiotherapist	Knowledge, awareness of patients receiving platinum-based chemotherapy, availability of resources, poor communication between disciplines and staff shortages.
Clinical psychologist	Team members involvement, resources and patient’s compliance.
Occupational therapist	Adequate training on ototoxicity.
Paediatric oncologist	Resources and lack of audiologists in state hospitals.
Nurse	Knowledge, staff commitment, training, resources and time.
Paediatrician	Shortage of audiologists.

Regression analysis revealed a statistically significant positive relationship (*p* = 0.002) between knowledge and practice, indicating that participants’ knowledge influenced their practices.

## Discussion

The current study revealed that 95.0% of the participants studied in South Africa, with 36.0% (*n* = 26) indicating not having studied or received training on the ear, hearing and ototoxicity. Furthermore, 54.0% (*n* = 34) of the participants indicated that the training or studies they received were inadequate. These findings suggest that most healthcare professionals educated in South Africa felt inadequately prepared to manage a patient receiving ototoxic medication. Inadequacy in the workplace can have serious consequences on healthcare professionals and patients. A study conducted by Odland et al. (2014) showed that a sense of inadequacy can conflict with the healthcare professionals’ professional identity, potentially prompting a shift in how they perceive their responsibilities. Consequently, the traditional role, centred on providing excellent care, might face uncertainty. The competency gap could be linked to an education gap (Odland et al., 2014) and underscores the necessity for academic training to equip healthcare professionals with comprehensive knowledge of platinum-induced ototoxicity before entering their professional roles. A comparison of the healthcare professionals’ knowledge of ototoxicity to that of its content in the different curriculums was, however, beyond the scope of the current study. The current study finding although suggests that more content and training in the area of ototoxicity should be included in the curriculum of undergraduate audiology programmes and in-service training. This suggestion is supported by previous literature findings. A scoping review conducted by Moodley et al. (2021) revealed a need to educate doctors on ototoxicity and audiologists on the pharmacology of ototoxic medication. A study by Khoza-Shangase and Masondo (2020) investigating the audiological practices for ototoxicity assessment and management in South Africa showed that there were significant gaps in audiologists’ knowledge of ototoxicity monitoring. In the study conducted by Paken et al. (2020), 82.0% of the participants, consisting of 7 oncologists, 9 nurses and 13 pharmacists, recognised audiologists as part of the oncology team, but the chemotherapy protocol did not include ototoxicity monitoring, nor was there an implementation of an ototoxicity monitoring programme.

Because of a lack of training and studies in ototoxicity during undergraduate studies, healthcare professionals turned to alternative educational platforms, with 44.0% of the participants reporting having received training through in-service training, postgraduate studies, orientation and on-the-job training. The results show another dimension to education and training, which could positively impact the appropriate management of patients receiving platinum-based chemotherapy drugs. Studies conducted over time have consistently shown a lack of clear understanding among patients and professionals regarding ototoxicity monitoring, thus highlighting the need for ongoing development and training in this area (Khoza-Shangase & Masondo, 2020; Moodley et al., 2021). Such initiatives involve creating ototoxicity monitoring guidelines explicitly tailored for South Africa and providing comprehensive training for doctors and audiologists to ensure adherence to these guidelines (Moodley et al., 2021). An earlier study by Wium and Gerber (2016) has also expressed similar sentiments. The study further stated that healthcare professionals who require more knowledge on ototoxicity would benefit from additional support, such as Continuing Professional Development (CPD) activities tailored to this area (Wium & Gerber, 2016). Healthcare professionals should attend CPD activities focussing on risk factors of ototoxicity, symptoms, side effects of ototoxicity and components of an ototoxicity monitoring programme.

The apparent inadequacy in education and training in ototoxicity is evident in the participants’ knowledge of the risk factors of ototoxic hearing loss. Twenty-seven per cent of participants indicated that they did not know if there were risk factors associated with ototoxic hearing loss in children receiving platinum-based chemotherapy. In the current study, the overall scores of participant’s knowledge of the associated risk factors of ototoxicity caused by platinum-based drugs were calculated to be a moderate achievement. This potentially suggests that their understanding of specific risk factors is uncertain, which aligns with participants’ feedback regarding insufficient training in this field. However, knowledge of the risk factors and symptoms of ototoxic hearing loss is crucial for identifying patients susceptible to ototoxicity and implementing appropriate management strategies to reduce its impact (Paken et al., 2020).

With regard to knowledge of the symptoms associated with ototoxic hearing loss, 20.3% of the participants identified dizziness as a symptom, while 51.0% indicated tinnitus as an associated symptom of ototoxic hearing loss. Overall knowledge scores on the symptoms of ototoxicity caused by platinum-based chemotherapy drugs indicated an elementary achievement according to the Department of Basic Education protocols for assessments. These findings are similar to a study conducted by de Andrade et al. (2009), which demonstrated that oncologist participants possessed basic knowledge about symptoms of ototoxic hearing loss and the physical alterations to the auditory system following the administration of ototoxic medication. Comparable results emerged from a later study in Gauteng, indicating that 80.0% of oncologists exhibited some understanding of hearing-related symptoms caused by chemotherapy, and 20.0% were not fully aware of the symptoms encountered by patients using ototoxic drugs (Khoza-Shangase & Hajat, 2009). Knowledge of the symptoms and risk factors enables the early identification of patients susceptible to ototoxic hearing loss, facilitating timely detection of any hearing impairment. Early detection and intervention of acquired hearing loss are critical in children, as hearing loss causes delayed language development, varied emotional regulation and disrupted social engagement and psychosocial development (Podury et al., 2023).

Sixty per cent of participants indicated that ototoxicity is a side effect of platinum-based chemotherapy drugs, while only 18.9% and 10.8% indicated that hyperacusis and aural fullness were side effects, respectively. Hyperacusis is a debilitating condition frequently linked with hearing loss induced by medication (Salvi et al., 2021). Another study indicated that injuries to the auditory system caused by drugs can manifest in several forms, including tinnitus, hearing loss, hyperacusis, sensation of fullness in the ears, dizziness and vertigo (Ganesan et al., 2018). The overall knowledge scores on the side effects of ototoxicity caused by platinum-based chemotherapy drugs indicated an elementary achievement. The current study findings differ from that of Paken et al. (2020), who indicated that all participants were aware of the side effects of certain chemotherapy drugs and that of Wium and Gerber (2016), who reported that slightly more than half of the participants (53.0%) attained a score of ≥80%, indicating a sufficient level of understanding of ototoxic medications and their associated side effects. However, the current study findings are consistent with that of Bora et al. (2020), which revealed that only 36.0% of medical doctors strongly agreed that chemotherapy drugs are known to cause permanent hearing loss. Understanding platinum-based chemotherapy’s side effects and risk factors is essential for educating the patient during pre-treatment counselling or subsequent visits, enabling the early detection of ototoxicity (Paken et al., 2020). The responsibility of early detection of ototoxicity is not the audiologist’s sole responsibility; however, a collaboration between multiple disciplines is part of the ototoxicity monitoring programme. Therefore, it is imperative for healthcare professionals treating children on platinum-based chemotherapy drugs to know their roles and the roles of other healthcare providers and work together in managing ototoxicity in this patient population because, throughout one’s life, hearing is crucial for language development, communication and building relationships. Early hearing loss in childhood can lead to negative psychosocial effects, while in adulthood, it may contribute to increased morbidity.

Sixty-eight (92.0%) participants indicated that the audiologist’s role within the ototoxicity monitoring programme was vital. Most healthcare professionals correctly identified the roles of audiologists, their roles and the roles of other healthcare professionals within the ototoxicity monitoring team. The current study, therefore, reflects similar findings to that of Paken et al. (2020), which indicated that most oncologist participants referred the patients to audiologists and all of the audiologists were aware of their roles of conducting audiological assessments in an ototoxicity monitoring programme. Although the current study presents a favourable outcome, it is worth noting that 44 (67.0%) participants were unsure whether the hospital (their current place of employment) had an established ototoxicity monitoring programme, which could possibly explain the lack of or the minimal number of referrals. Hence, training and education on ototoxicity monitoring programmes are essential.

It is important to highlight that 27.0% of the participants in the current study were unaware of the components of the ototoxicity monitoring programme. Furthermore, only 10.8% recognised baseline assessment as one of its elements, while 4.1% selected treatment adjustments based on changes in hearing thresholds, and 5.4% included post-treatment completion follow-up as part of the programme. Additionally, 43 (73.0%) participants stated that they did not know how long the ototoxicity monitoring programme continued post-treatment. In the current study, the overall scores of participants’ understanding of the components of the ototoxicity monitoring programme were classified as ‘Not Achieved’. These results are concerning because, according to HPCSA (2018), children undergoing platinum therapy require follow-up sessions at 3, 6 and 12 months post-treatment. The inclusion of long-term follow-up assessments is crucial for monitoring the delayed onset of hearing loss that may manifest over time (Brooks & Knight, 2017), as cisplatin can be retained in the cochlea for up to 20 years (Breglio et al., 2017). Adhering to ototoxicity monitoring guidelines is imperative for healthcare professionals to ensure timely diagnoses of ototoxic hearing loss. Childhood hearing loss does not just affect language development, it also impacts literacy, self-esteem and social skills (WHO, 2016). When left untreated, it can result in academic struggles, limiting employment prospects in the future (WHO, 2016).

Difficulty in communication can lead to long-term emotional issues such as isolation, loneliness and depression (WHO, 2016). Families of children with hearing impairments face unique challenges, including heightened stress levels, increased financial burdens and communication obstacles with their children (WHO, 2016). Moreover, untreated hearing loss can hinder the social and economic progress of communities and countries at large (WHO, 2016). This observation further underscores the requirement for training initiatives, such as in-service training programme, to educate healthcare professionals about the ototoxicity monitoring programme. A scoping review by Moodley et al. (2021) showed that studies conducted over the years consistently suggest that monitoring ototoxicity remains unclear to professionals, thus highlighting the ongoing need for further interprofessional education in this area. Interprofessional education and collaborative practice can have a substantial impact on addressing numerous challenges encountered by health systems (WHO, 2010). For health workers to effectively collaborate and enhance health outcomes, individuals from diverse professional backgrounds must be given opportunities to learn about, from and with each other (WHO, 2010). Following nearly 50 years of investigation, ample evidence suggests that interprofessional education facilitates effective collaborative practice, which optimises health services, strengthens health systems and enhances health outcomes (WHO, 2010). Implementing interprofessional education and collaborative practice brings about a transformation in how health workers interact with each other to provide care, representing one of the advantages of these strategies.

As per the American Academy of Audiology (AAA) position statement in 2009, the team members involved in the ototoxic monitoring programme encompass doctors, nurses, pharmacists, clinical psychologists, physiotherapists and speech therapists. Collaboration among patients, families and the healthcare team is required to ensure the programme’s success. Current study findings revealed that not all participants involved in the ototoxicity monitoring programme adopted a team approach. The lack of collaborative working relationships among the teams managing patients on ototoxic medications is not a recent discovery. Khoza-Shangase and Jina (2013) discovered that South African general practitioners, despite being aware of ototoxicity, its symptoms and the availability of audiological services, did not incorporate these services into their patient care. The study concluded that general practitioners prioritise the patient’s diagnosis over less visible side effects such as ototoxicity (Khoza-Shangase & Jina, 2013). In another investigation, Wium and Gerber (2016) found that practitioners refrain from referring to audiologists because of time constraints and insufficient knowledge about ototoxicity signs and symptoms from both the practitioner and the patient. In a study conducted by Ehlert et al. (2022), healthcare professionals identified the referral system as the most prominent challenge, and this was attributed to them not prioritising hearing loss. Oncology units claim that cancer diagnosis, advanced disease, other oncologic emergencies and emotional, financial and physical constraints are prioritised (Carrera et al., 2018; Oun et al., 2018).

Regarding audiologists’ ototoxicity monitoring practices, all audiologist participants (*n* = 4) reported conducting otoscopy, tympanometry, acoustic reflex threshold testing and pure tone audiometry for baseline and follow-up assessments. However, only 50.0% (*n* = 2) conducted high-frequency audiometry at baseline and 25.0% excluded distortion product otoacoustic emission test from the follow-up assessment. According to HPCSA (2018) guidelines, air conduction pure tone audiometry should be conducted at 250 Hz – 12 500 Hz for baseline assessments, and Distortion Product Otoacoustic Emissions (DPOAEs) should be included in the follow-up assessment. The ototoxicity monitoring protocol for children is different from that of adults and requires consideration of the child’s age and wellness. In cases where children are old enough but are unwell to engage in behavioural audiometry, adaptations to the testing protocol need to be considered. According to HPCSA (2018) guidelines, tests such as distortion product otoacoustic emissions and immittance, immittance audiometry and a selected number of pure tone and high-frequency thresholds (assess 4 kHz, 6 kHz, 8 kHz, 10 kHz, 12.5 kHz) should be prioritised. In cases where children are too young to cope with behavioural audiometry, diagnostic DPOAEs should be conducted at high frequencies (4000 Hz and above) (HPCSA, 2018). With regard to monitoring evaluations, for children younger than 5 years old, DPOAEs should be conducted (HPCSA, 2018). If DPOAEs are absent, then an auditory brainstem response test or auditory steady-state response test should be conducted (HPCSA, 2018).

It is essential to include testing for extended high frequencies in ototoxicity monitoring, as platinum-based drugs typically impact higher frequencies before affecting lower frequencies (Edward et al., 2015). Failure to include extended high frequencies in ototoxicity monitoring may result in overlooking hearing loss, as a South African cohort study by Paken et al. (2023) showed a notable correlation (*p* < 0.05) between changes in hearing and cisplatin dosage, particularly in frequencies of 9000 Hz and higher. Audiologists may encounter challenges leading to the improper implementation of ototoxicity monitoring protocols. A study conducted by Khoza-Shangase and Masondo (2020) revealed that while more than two-thirds of the participants are involved in ototoxicity monitoring and management, their practices do not conform to international standards or the national HPCSA guidelines for assessing and managing patients on ototoxic medications.

Emotional counselling is also just as crucial to ensuring the emotional and mental well-being of children diagnosed with cancer. The current study showed that only 50.0% of psychologist participants (*n* = 2) offered counselling to the patient’s families. Early and continuous assessment of the mental health needs of parents and caregivers of children with cancer is crucial. Previous studies by Barrera et al. (2004), Sloper (2000), Pai et al. (2007) and Kazak and Baxt (2007) have shown that emotional challenges faced by a parent can potentially disrupt the cancer treatment of the child, affect their ability to provide support and care and pose a threat to family functioning and stability over time. Facilitating access to suitable interventions for parents and caregivers is essential to enhance the well-being of parents, the affected child and the overall family (Barrera et al., 2004; Kazak et al., 2007; Pai et al., 2007; Sloper, 2000).

Twelve ENTs (87.5%) indicated that the criterion for referral for an audiological assessment was all children receiving platinum-based chemotherapy drugs, while only 35.7% (*n* = 5) included repeated doses as a criterion to refer for audiological assessment. Cumulative dose is a risk factor for ototoxic hearing loss (Landier et al., 2014). This finding suggests that children who received repeated doses of cisplatin were not referred for audiological assessment. This is concerning as literature showed that the severity of hearing loss is directly correlated with the cumulative dose of cisplatin (Bertolini et al., 2004). Therefore, all patients on cisplatin should be monitored. Although 63.2% of nurses mentioned difficulty hearing as a criterion for referring individuals for audiological assessment, only 36.8% considered difficulty communicating and frequent requests for repetition. Tinnitus and dizziness were utilised as criteria by only 26.3% and 21.1% of nurses, while 15.8% used hypersensitivity to sound as a criterion. These findings are concerning, as tinnitus, dizziness, hypersensitivity to sound, difficulty communicating and frequent requests for repetition are indicative signs and symptoms of hearing loss. Although most nurse participants understood that their role included monitoring signs and symptoms of hearing loss, there are gaps in their knowledge of the symptoms. With a lack of comprehensive understanding of symptoms, patients presenting with symptoms of hearing loss could be missed by nurses.

The success of the ototoxicity monitoring programme extends beyond knowledge. Various factors contribute to adherence to the ototoxicity monitoring programme. Adherence to ototoxicity monitoring is influenced by factors such as service accessibility, health promotion and an interdisciplinary approach, as noted by Ganesan et al. (2018). Results of the current study indicate that lack of time because of workload influences healthcare professionals’ practice in managing ototoxicity in children. Pillay (2020) showed that nurses within the South African public health sector expressed significant dissatisfaction with their compensation, workload and available resources. These factors can potentially impact the implementation of an ototoxicity monitoring programme (Pillay, 2020). Shortage of staff was another contributing factor reported by participants, and these findings are in keeping with Boniol et al. (2022), who indicated that by 2023, there will be a shortage of 10 million healthcare professionals. Across sub-Saharan Africa, a significant weakness in health systems is the insufficient availability of human resources. WHO (2022) indicated that Africa has less than one health worker for every 1000 people, a stark contrast to the 10 per 1000 in Europe (Fonn et al., 2011). Barron and Padarath (2017) observed that health challenges in South Africa are exacerbated by an uneven distribution of health professionals between the private and public sectors and an unequal distribution of public sector health professionals among different provinces. In 2015, KwaZulu-Natal Department of Health audit outcomes revealed that a lack of availability of doctors and professional staff contributed to the healthcare challenges in the province (Dlamini, 2015). Manyisa and Aswegen (2017) noted that the inadequacy of health workers and poor working conditions lead to physical and psychosocial exhaustion and sometimes worsen healthcare personnel’s medical conditions. Pillay et al. (2020) stated that, under the best-case scenario estimation, a gap of approximately 2800 professionals exists between supply and demand. The probability of South Africa’s Burden of Illness and Disability (BOID) being linked to communication disorders, hearing impairment, balance/vestibular issues or swallowing disorders is significant (Pillay et al., 2020). The current study revealed that a lack of medical equipment was a factor that influenced the practices of healthcare professionals. This finding correlates with a study by Moyimane et al. (2017), which reflected that the shortage of medical equipment had dire consequences on patients, the staff, the hospital and the functioning of the health system. Moreover, a study by the Human Science Research Council in 2013 indicates that the public sector is not delivering rehabilitation services effectively, efficiently or equitably (Louw et al., 2023). The current study’s findings show that human resource shortages, financial constraints, human resource development, lack of equipment and timeous access to rehabilitation services remain a challenge. Additional challenges, such as team members’ insufficient knowledge about ototoxicity, inadequate communication among team members and a lack of training in ototoxicity, can further impede the proper management of ototoxicity within the team (Khoza-Shangase & Masondo, 2020).

While the current study shed light on various contextual barriers and identified areas of improvement regarding the knowledge base of healthcare professionals, it is important to acknowledge both its limitations and strengths. The limitations of the study are as follows:

- The number of participants per fraternity was small, which affected the generalisation of findings per fraternity. This could impact the interpretation of the results per fraternity.
- Data were obtained using a structured questionnaire. A limitation to this is that responses lacked depth and were self-reported. This inherent limitation suggests that the data may not fully capture the complexity of the participants’ responses.
- With the use of self-administered questionnaires, socially influenced responses cannot be overlooked. Participants could state what they think is the truth or what they think the researcher wants; people are not always aware of their thoughts, feelings, attitudes and opinions. This could affect the reliability of the results.
- Research bias cannot be discounted. Questionnaires were left with participants to complete. The possibility of responses being researched rather than truly reflecting the participants’ genuine understanding of ototoxicity caused by platinum-based drugs cannot be ignored.
- The researcher relied on HODs to distribute the questionnaire and information pamphlets and remind participants to complete the questionnaire. The possibility of HODs forgetting to distribute and remind timeously cannot be overlooked. This could have affected the response rate as well as the participants’ knowledge of ototoxicity being reinforced in the area of ototoxicity.
- The researcher viewed the various professions with diverse scopes with the same lens, and this may have influenced the data obtained.

Strengths of the study are as follows:

- This study obtained an 82% response rate. A response rate of ≥ 80% is desired as the data obtained represents the population of interest (Fincham, 2008). Although the response rate per healthcare fraternity was low, the overall response rate was high. This allows the results to represent the ototoxicity monitoring team as a whole.
- A purposive sampling technique was used. The researcher selected participants who were most pertinent to answer the research question.
- The questionnaire underwent an evaluation process where an expert in the area of ototoxicity critically analysed the research tool.
- This study sites included public hospitals offering comprehensive oncology services to paediatrics making it suitable to answer the research question.
- The survey consisted of mainly close-ended and limited open-ended questions. This allowed for the comprehensive acquisition of both qualitative and quantitative information to be collected from a diverse group of healthcare professionals.

The clinical inferences the current study offers encompass reviewing and improving the undergraduate curriculum are necessary for education and training on ototoxicity for disciplines part of the ototoxicity monitoring team. Attention should be placed on enhancing the understanding of the ear, hearing loss, ototoxic medications and their side effects, as well as symptoms and risk factors associated with hearing impairment. This could help to sufficiently prepare pharmacists, audiologists, occupational therapists, physiotherapists, clinical psychologists, paediatric oncology nurses and doctors to holistically manage paediatrics receiving platinum-based chemotherapy. Audiologists should develop regular and organised training programmes using the HPCSA’s guidelines on ototoxicity management. This could assist with enhancing the knowledge and improving the practices of all healthcare professionals within the ototoxicity monitoring programme. The training could be conducted annually during staff meetings. This could influence the way healthcare professionals manage paediatrics on platinum-based chemotherapy nurses may monitor signs and symptoms and make the necessary referrals. Pharmacists may raise awareness of ototoxicity and may recommend otoprotective agents. Clinical psychologists may appropriately manage the mental well-being of patients on platinum-based chemotherapy appropriately. Enhancement in their knowledge may help them differentiate between ototoxic and mental symptoms. Occupational therapists could aptly manage the patients’ return to education. Physiotherapists could work closely with audiologists to provide inter-professional vestibular rehabilitation, thus optimising patient care.

Facilities offering oncology services to paediatrics should have readily accessible resources, such as pamphlets, posters and booklets regarding ototoxicity for patients, their families and staff. The Department of Health should employ more audiologists to improve the accessibility of ototoxicity monitoring services. With more staff available, service delivery, specifically ototoxicity monitoring, such as bi-weekly audiological monitoring, can be improved. Audiologists should actively advocate for their profession by disseminating more information about audiology. This effort should aim to enhance colleagues’ understanding of the breadth of audiology practice. The Department of Health should prioritise rehabilitation by ensuring even distribution of resources, improving human shortages and access to rehabilitation services. This ensures that adequate support is available for monitoring and managing ototoxicity, particularly in high-risk populations such as paediatric patients. Policy formulation and implementation on ototoxicity in facilities offering platinum-based chemotherapy treatment are crucial as this can assist in providing a standardised approach to the prevention, monitoring and management of ototoxicity. Policy formulation and implementation can ensure that all healthcare professionals follow consistent guidelines, which improves the quality of care and reduces variability in patient outcomes. Effective policies help mitigate the risks associated with ototoxic medications. Policies help ensure that healthcare practices comply with regulatory requirements and standards. This can include guidelines on the safe use of ototoxic medications and protocols for reporting adverse effects. Policies can provide clear guidance for patients and their families on what to expect during treatment with ototoxic medications. This includes information on potential side effects, monitoring procedures and available support resources.

Currently, there are limited South African studies on ototoxicity because of platinum-based chemotherapy in the paediatric population. Further research should investigate at a national level the knowledge and practices of healthcare professionals on ototoxic hearing loss in children receiving platinum-based chemotherapy. Further studies should investigate the success of the HPCSA ototoxicity monitoring protocol. The current study found that there were various contextual barriers that influenced healthcare professional’s practices in managing children with ototoxicity. Thus, these factors can impact the success of the ototoxicity monitoring protocols at facilities managing children on platinum-based chemotherapy treatment. According to Fernandez et al. (2023), modifications to existing ototoxicity monitoring protocols should consider factors such as patients, the care team and institutions. Future research should focus on examining the curriculum requirements for various healthcare professions involved in ototoxicity monitoring programme. This includes evaluating the integration of ototoxicity-related content into their educational frameworks. Additionally, it is important to investigate healthcare professionals’ exposure to CPD opportunities and assess the quality of CPD training they receive. Understanding these aspects will provide insights into the effectiveness of current training and identify potential areas for improvement, ensuring that healthcare professionals are adequately prepared to manage and monitor ototoxicity in their practice. The current study opens avenues for longitudinal studies to assess the impact of improved training and practices on patient outcomes. Further research is required to assess the efficacy and efficacy of ototoxicity programmes, and some site-specific considerations may be needed.

## Conclusion

The present study findings demonstrate a clear correlation between healthcare professionals’ practices and their level of knowledge. This study revealed that 45 participants (60.8%) identified ototoxicity as a side effect of platinum-based chemotherapeutic drugs, 43 participants (58.1%) identified dose, duration and mode of administration as risk factors, 43 participants (72.9%) did not know the duration of an ototoxicity monitoring programme post-treatment. These findings underscore the importance of improving the knowledge base of healthcare professionals involved in ototoxicity monitoring programme to enhance their practices effectively. This supports the need for education and training initiatives to be implemented at facilities providing oncology services to paediatric patients, aimed at bolstering healthcare professionals’ efficiency in this domain. Some of the critical findings of the current study included knowledge gaps among some healthcare professionals, such as symptoms related to hearing, two-thirds of participants did not know whether their workplace had an ototoxicity monitoring programme, and 27.0% did not accurately identify all the components of an ototoxicity monitoring programme. Seventy-three per cent indicated not knowing the duration of an ototoxicity monitoring programme post-treatment, while only 17.0% accurately indicated that the ototoxicity monitoring programme continues for up to 6 months following cessation of treatment. Not all participants fully adhered to HPCSA guidelines on ototoxicity monitoring. Despite these identified gaps, the current study revealed some positive outcomes. Healthcare professionals acknowledged the significance of the audiologist’s role, correctly identifying their involvement within the ototoxicity monitoring programme. However, the number of participants per fraternity was small, which affected the generalisation of findings per fraternity. Data were obtained using a structured questionnaire; the responses lacked depth and were self-reported; future research should consider the use of focus groups or interviews to probe participants’ responses further. Key clinical implications of the current study include the need to review and improve education and training on ototoxicity within undergraduate syllabi. Audiologists should establish regular training programme on ototoxicity, which could enhance the management of paediatric patients on platinum-based chemotherapy. Nurses should monitor symptoms and make necessary referrals. Pharmacists should raise awareness and recommend otoprotective agents. Clinical psychologists should manage the mental well-being of patients on chemotherapy. Occupational therapists should assist in the patients’ return to education and physiotherapists should collaborate with audiologists for vestibular rehabilitation. Policy formulation and implementation on ototoxicity as this can assist in providing a standardised approach to the prevention, monitoring and management of ototoxicity. Effective policies help mitigate the risks associated with ototoxic medications. Additionally, facilities offering oncology services to paediatrics should have readily accessible resources. The Department of Health should prioritise rehabilitation by ensuring even distribution of resources, improving human resources and access to rehabilitation services. Further research should investigate the knowledge and practices of healthcare professionals on ototoxic hearing loss in children receiving platinum-based chemotherapy at a national level, as well as to investigate the feasibility of using HPCSA guidelines.
